# LRRK2 regulates actin assembly for spindle migration and mitochondrial function in mouse oocyte meiosis

**DOI:** 10.1093/jmcb/mjab079

**Published:** 2021-12-16

**Authors:** Zhen-Nan Pan, Jing-Cai Liu, Jia-Qian Ju, Yue Wang, Shao-Chen Sun

**Affiliations:** College of Animal Science and Technology, Nanjing Agricultural University, Nanjing 210095, China; College of Animal Science and Technology, Nanjing Agricultural University, Nanjing 210095, China; College of Animal Science and Technology, Nanjing Agricultural University, Nanjing 210095, China; College of Animal Science and Technology, Nanjing Agricultural University, Nanjing 210095, China; College of Animal Science and Technology, Nanjing Agricultural University, Nanjing 210095, China

**Keywords:** actin, spindle, LRRK2, oocyte, meiosis

## Abstract

Leucine-rich-repeat kinase 2 (LRRK2) belongs to the Roco GTPase family and is a large multidomain protein harboring both GTPase and kinase activities. LRRK2 plays indispensable roles in many processes, such as autophagy and vesicle trafficking in mitosis. In this study, we showed the critical roles of LRRK2 in mammalian oocyte meiosis. LRRK2 is mainly accumulated at the meiotic spindle periphery during oocyte maturation. Depleting LRRK2 led to the polar body extrusion defects and also induced large polar bodies in mouse oocytes. Mass spectrometry analysis and co-immunoprecipitation results showed that LRRK2 was associated with several actin-regulating factors, such as Fascin and Rho-kinase (ROCK), and depletion of LRRK2 affected the expression of ROCK, phosphorylated cofilin, and Fascin. Further analysis showed that LRRK2 depletion did not affect spindle organization but caused the failure of spindle migration, which was largely due to the decrease of cytoplasmic actin filaments. Moreover, LRRK2 showed a similar localization pattern to mitochondria, and LRRK2 was associated with several mitochondria-related proteins. Indeed, mitochondrial distribution and function were both disrupted in LRRK2-depleted oocytes. In summary, our results indicated the critical roles of LRRK2 in actin assembly for spindle migration and mitochondrial function in mouse oocyte meiosis.

## Introduction

Mammalian oocyte meiotic maturation is characterized as asymmetric division that produces a large metaphase II (MII)-arrested oocyte and a small polar body, which is different from symmetric cell division that occurs in mitosis. This process is critical for fertilization and early embryo development, and the failure of asymmetric division will result in low oocyte quality (Maro and Verlhac, 2002; Sun and Schatten, 2006). The oocyte meiotic spindle forms in the central cytoplasm after germinal vesicle breakdown (GVBD) and its migration to the cortex is essential for asymmetric division (Brunet and Verlhac, 2011). Cytoplasmic actin plays an important role in spindle migration in the metaphase I (MI) stage (Yi et al., 2013a). A variety of small GTPases and kinases could influence actin-mediated spindle migration. RAB35 is a member of Ras-related GTP-binding proteins (RABs) which belong to a superfamily of small GTPases, and could affect spindle migration through actin assembly in mouse oocyte meiosis (Zhang et al., 2019a). RAB11 could transport the actin nucleation factor for actin dynamics, which further affect the meiotic spindle migration in mouse oocytes (Holubcova et al., 2013). Besides, other subfamilies of small GTPases such as Ras homolog family member (Rho) and ADP-ribosylation factor are also involved in actin dynamics for spindle positioning of oocytes (Duan et al., 2018a, b). For kinases, Rho-kinase (ROCK) is reported to regulate actin-dependent meiotic spindle migration (Duan et al., 2014), while protein kinase C mu (PKC mu) affects the phosphorylation level of cofilin, which further regulates cytoplasmic actin distribution and spindle positioning (Zhang et al., 2019b). In addition to regulating spindle migration, actin also plays a vital role in maintaining the distribution and function of mitochondria during oocyte meiotic maturation (Duan et al., 2020). Mitochondria are the important organelle generating cellular energy, which is a critical determinant of the oocyte developmental potential (Cecchino et al., 2018). Small GTPases and kinases affect mitochondrial function by the regulation of actin filaments. Mitochondrial Rho GTPases (MIROs) could coordinate actin-dependent mitochondrial transport and distribution (Lopez-Domenech et al., 2018). The RAB7 GTPase regulates actin dynamics for dynamin-related protein 1 (DRP1)-mediated mitochondrial function in mouse oocyte meiosis (Pan et al., 2020). Moreover, the disruption of protein kinase ROCK activity results in actin-mediated spindle migration and mitochondrial distribution defects (Duan et al., 2014). Emerging evidence indicates that the spindle-peripheral actin is important for the accumulation of mitochondria in this region and spindle migration (Duan et al., 2020). Therefore, actin plays an essential role in the meiotic maturation of oocytes for spindle migration and mitochondrial function.

Leucine-rich-repeat kinase 2 (LRRK2) includes several domains implicated in protein‒protein interactions and has a central region consisting of a Ras-of-complex (ROC) GTPase domain and a kinase domain connected via a C-terminal of the ROC (COR) domain (Kuwahara and Iwatsubo, 2020). Therefore, LRRK2 is a protein with both GTPase and kinase functions. LRRK2 is associated with a diverse set of cellular functions and signaling pathways (Wallings et al., 2015), including cytoskeletal dynamics (Chan et al., 2011), mitochondrial function (Wang et al., 2012), vesicle trafficking (Lanning et al., 2018), and autophagy (Alegre-Abarrategui et al., 2009). LRRK2 plays an important role in actin assembly. The neurons in LRRK2^−^^/^^−^ mice show a significant reduction in the size of the F-actin-positive area in the filopodia (Parisiadou et al., 2009). LRRK2 could affect actin polymerization/depolymerization since LRRK2 depletion decreases the F-actin/G-actin ratio in microglia (Kim et al., 2018). Coexpression with LRRK2 increases RAC1 activity, which could modulate actin cytoskeletal dynamics in HEK293 cells (Chan et al., 2011). Moreover, LRRK2 could regulate mitochondrial dynamics and function through direct interaction with dynamin-like protein 1 (DLP1) via its kinase activity (Wang et al., 2012). The percentage of fragmented mitochondria is elevated in LRRK2^−^^/^^−^ MEFs and the activity of citrate synthase has a remarkable reduction, indicating its roles in the mitochondrial structure (Toyofuku et al., 2020). Moreover, inhibition of LRRK2 causes DRP1-mediated mitochondrial fission and induces mitophagy in SH-SY5Y cells (Saez-Atienzar et al., 2014). Although LRRK2 is reported to be involved in various biological processes in somatic cells, the function of LRRK2 during mouse oocyte meiosis remains uncertain.

In our study, by the knockdown approach and mass spectrometry (MS) analysis, we confirmed that LRRK2 was related to actin-related spindle migration and mitochondrial function during mouse oocyte meiotic maturation.

## Results

### Expression and distribution of LRRK2 during oocyte meiosis

**Figure 1:**
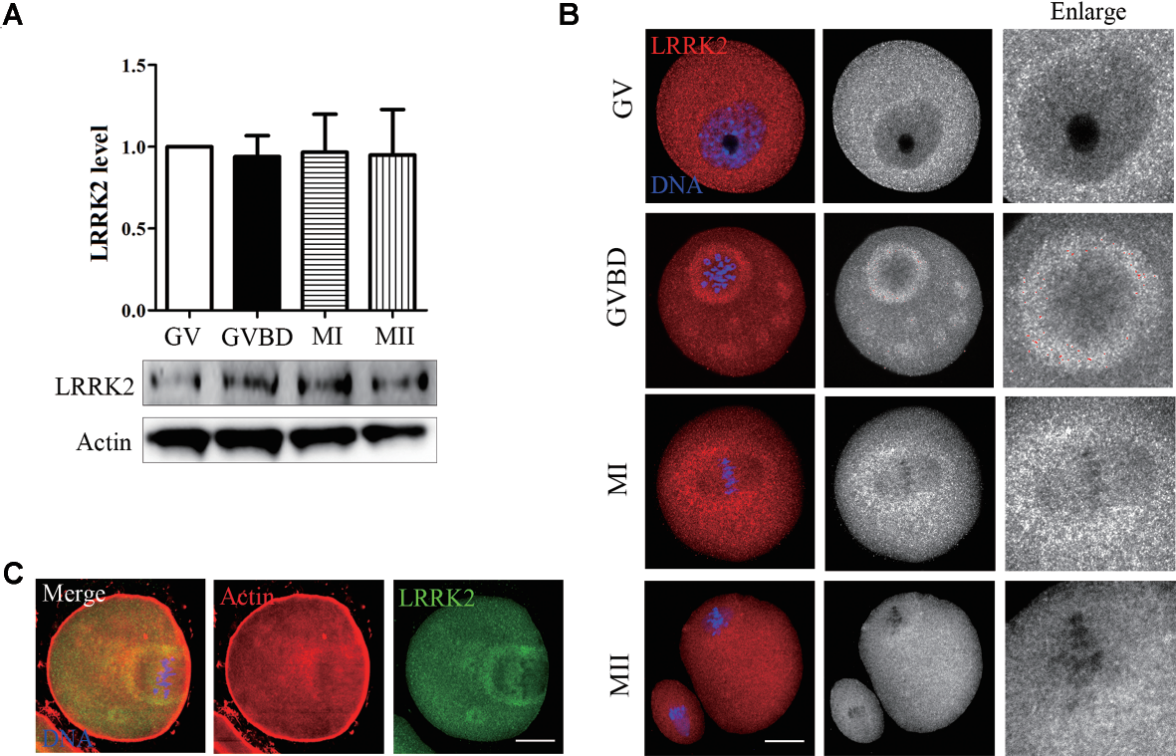
Expression and localization of LRRK2 in mouse oocyte meiosis. (**A**) The expression level of LRRK2 at different stages (GV, GVBD, MI, and MII) was examined by western blotting in mouse oocytes. (**B**) The localization of LRRK2 was certificated by LRRK2 antibody in mouse oocytes. LRRK2 dispersed in the cytoplasm at the GV stage and was mainly distributed around the spindle after GVBD. Red, LRRK2; blue, DNA; scale bar, 20 μm. (**C**) LRRK2 was colocalized with actin around the spindle in the MI-stage oocytes. Red, actin; green, LRRK2; blue, DNA; scale bar, 20 μm.

We first examined the expression level and distribution of LRRK2 at different stages of mouse oocyte meiosis. As shown in Figure 1A, LRRK2 was expressed in all stages of oocyte meiosis, including germinal vesicle (GV), GVBD, MI, and MII. Immunofluorescence staining results suggested that LRRK2 localized in the cytoplasm at the GV stage and was mainly concentrated around the spindle after GVBD (Figure 1B). Co-staining with phalloidin indicated that LRRK2 had a similar localization pattern to actin in the cytoplasm at the MI stage (Figure 1C). The distribution pattern of LRRK2 suggested the potential roles of LRRK2 in actin during oocyte maturation.

### LRRK2 is essential for mouse oocyte polar body extrusion

**Figure 2:**
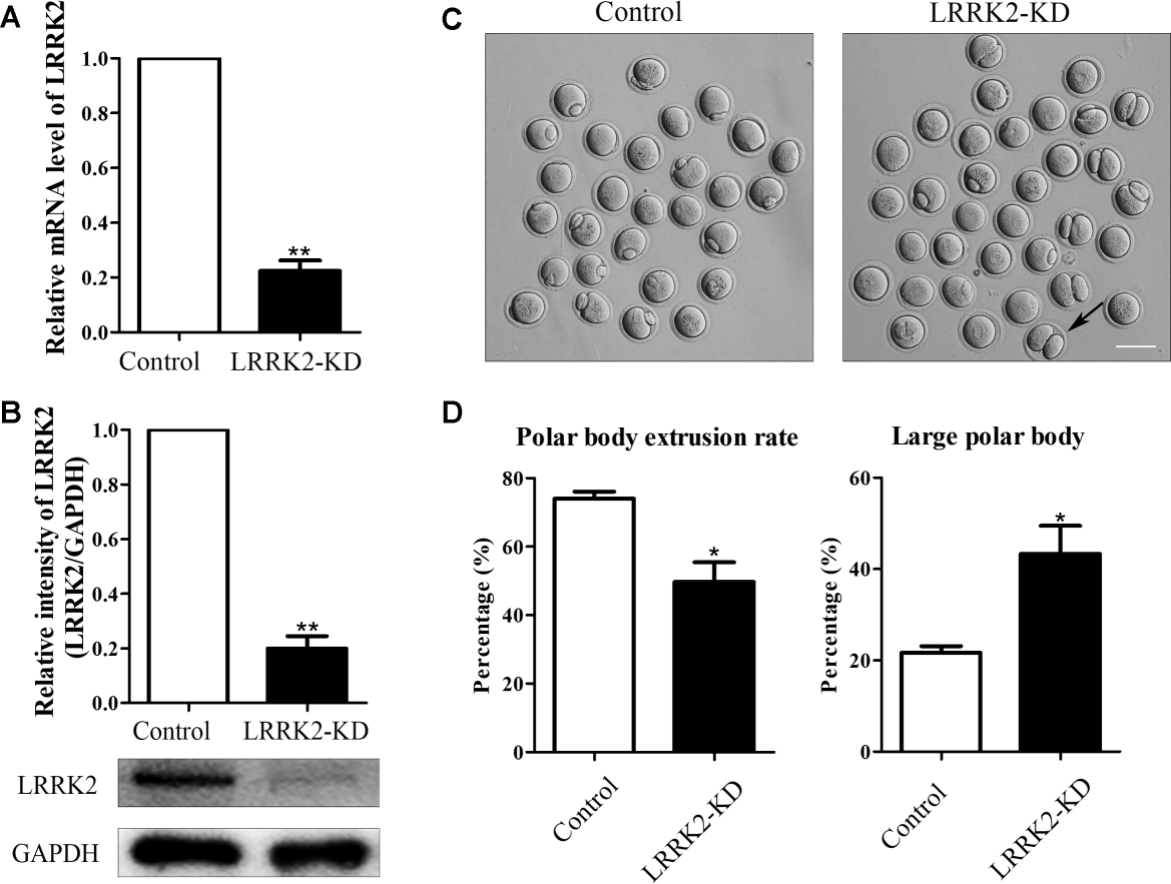
LRRK2 depletion affects mouse oocyte maturation. (**A**) The LRRK2 mRNA expression level was tested by RT-PCR in the LRRK2 siRNA-injected and control oocytes. (**B**) The LRRK2 protein expression level was examined by western blotting in the LRRK2 siRNA-injected and control oocytes. GAPDH served as the control. Band intensities were analyzed by Image J software. (**C**) LRRK2 depletion caused polar body extrusion defects and large polar bodies in mouse oocytes. The black arrow indicates an oocyte extruding a large polar body after 12 h culture. Scale bar, 100 μm. (**D**) The rate of polar body extrusion was lower, whereas the rate of the large polar body was higher in the LRRK2 siRNA-injected group compared with that in the control group. *P < 0.05 and **P < 0.01.

To investigate the roles of LRRK2 during oocyte maturation, we knocked down the expression of LRRK2 through siRNA injection. Oocytes were arrested in M2 medium with 3-isobutyl-1-methylxanthine (IBMX) for 24 h and then collected for testing the knockdown efficiency at both mRNA and protein levels. Real-time quantitative polymerase chain reaction (RT-PCR) results showed that siRNA injection led to a significant decrease of LRRK2 at the mRNA level (1 vs. 0.22 ± 0.04, *P* < 0.01; Figure 2A). In line with this, western blotting analysis showed that the protein expression level of LRRK2 was significantly reduced in oocytes (1 vs. 0.20 ± 0.05, *P* < 0.01; Figure 2B). Then, we examined first polar body extrusion in LRRK2 siRNA-injected oocytes. Our results indicated that LRRK2 depletion caused defects in both the first polar body extrusion and the asymmetric division, resulting in no polar body or big polar bodies (Figure 2C). Compared with control oocytes, there was a significant decrease of the polar body extrusion rate (74.00% ± 2.08%, *n* = 132 vs. 49.67% ± 5.78%, *n* = 144, *P* < 0.05; Figure 2D) and a significant increase of the large polar body (polar body diameter >1/3 of the oocyte diameter) rate in LRRK2 siRNA-injected oocytes (21.67% ± 1.45%, *n* = 101 vs. 43.33% ± 6.12%, *n* = 68, *P* < 0.05; Figure 2D). These results indicated that LRRK2 was important for mouse oocyte maturation.

### LRRK2 facilitates actin assembly for spindle migration in mouse oocytes

**Figure 3:**
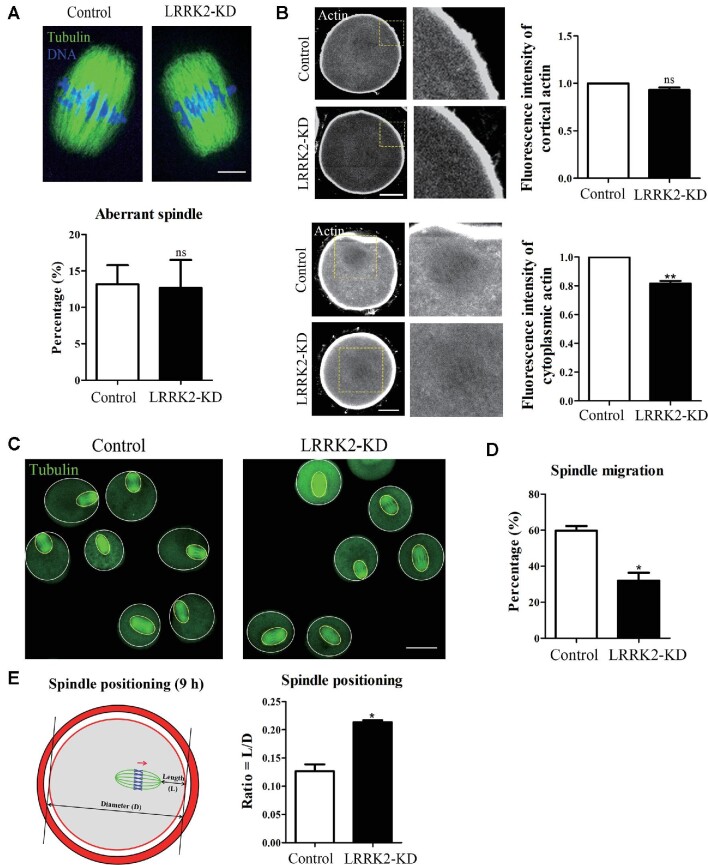
LRRK2 depletion affects cytoplasmic actin assembly for spindle migration in mouse oocytes. (**A**) Immunofluorescence staining showed that the spindle morphology of the LRRK2 siRNA-injected oocytes was normal and there was no difference in the percentage of aberrant spindle compared with the control oocytes. Green, tubulin; blue, DNA; scale bar, 10 μm. (**B**) LRRK2 depletion did not affect the fluorescence intensity of cortical actin but decreased that of cytoplasmic actin in the LRRK2 siRNA-injected oocytes. Gray, actin; scale bar, 20 μm. (**C**) Representative images of the spindle positioning in the control and LRRK2 siRNA-injected oocytes after 9 h culture. Green, tubulin; scale bar, 50 μm. (**D**) The meiotic spindles migrated under the cortex after 9 h culture in the control oocytes, while rates of spindle to the cortex were significantly decreased in the LRRK2 siRNA-injected oocytes. (**E**) Quantitative analysis of the extent of spindle migration showed that the *L*/*D* ratio was higher in the LRRK2 siRNA-injected oocytes than in the control oocytes. Red, actin; green, spindle; arrow, the direction of spindle migration. *P < 0.05, **P < 0.01, and ns, no significant difference.

Next, we examined the morphology of spindles and the fluorescence intensity of F-actin in LRRK2 siRNA-injected oocytes. We found that there was no significant difference for the abnormal spindles after LRRK2 siRNA injection (13.17% ± 2.62%, *n* = 29 vs. 12.67% ± 3.84%, *n* = 32, no significant difference; Figure 3A). Immunofluorescence staining results showed that the F-actin fluorescent signals at the cortical region in the LRRK2 siRNA-injected oocytes were not significantly different from that in the control oocytes (1, *n* = 30 vs. 0.93 ± 0.03, *n* = 27, no significant difference; Figure 3B); however, the fluorescent signals of cytoplasmic actin were significantly decreased in the LRRK2 siRNA-injected oocytes (1, *n* = 30 vs. 0.82 ± 0.02, *n* = 27, *P* < 0.01; Figure 3B). Cytoplasmic actin filaments are the key for meiotic spindle migration. We then explored the status of spindle migration after LRRK2 depletion. After 9 h culture, most spindles migrated to the cortex in control oocytes, while a large proportion of spindles remained at the center in the LRRK2 siRNA-injected oocytes (Figure 3C), which was validated by statistical analysis (59.67% ± 2.60%, *n* = 45 vs. 32.00% ± 4.36%, *n*=41, *P* < 0.05; Figure 3D). To quantify the extent of spindle migration, we regarded the oocyte diameter as *D* and the distance from the spindle pole to the oocyte cortex as *L*, and the *L*/*D* ratio represented the extent of spindle migration to the cortex. Our results indicated that the *L*/*D* ratio of the LRRK2 siRNA-injected oocytes was notably higher than that of the control oocytes (0.13 ± 0.01, *n* = 37 vs. 0.21 ± 0.01, *n*=33, *P* < 0.05; Figure 3E). These results indicated that LRRK2 affected cytoplasmic actin for spindle migration during mouse oocyte meiosis.

### LRRK2 regulates ROCK and cofilin for actin assembly in mouse oocytes

**Figure 4:**
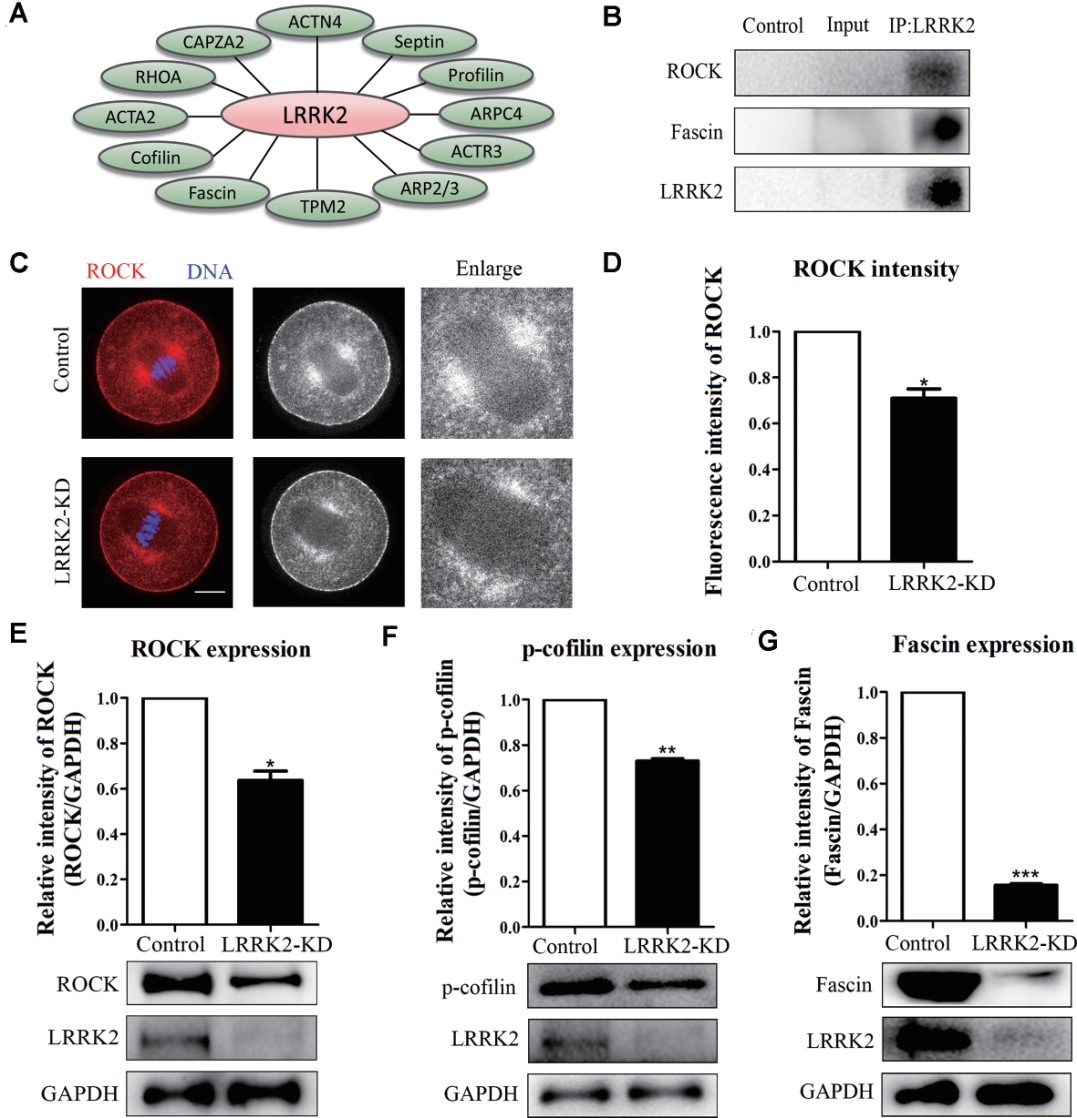
LRRK2 depletion affects the expression of ROCK‒cofilin and Fascin in mouse oocytes. (**A**) Screening actin-related proteins associated with LRRK2 by MS analysis. (**B**) Co-IP results showed that LRRK2 was correlated with ROCK and Fascin. (**C**) Immunofluorescence staining displayed the localization of ROCK in the control and LRRK2 siRNA-injected oocytes. Red, ROCK; blue, DNA; scale bar, 20 μm. (**D**) Statistical analysis showed that the fluorescence intensity of ROCK was significantly decreased at the spindle periphery after LRRK2 depletion. (**E**) ROCK protein expression was significantly decreased in the LRRK2 siRNA-injected oocytes. (**F**) The phosphorylation level of cofilin was significantly decreased in the LRRK2 siRNA-injected oocytes. (**G**) Fascin protein expression was significantly decreased in the LRRK2 siRNA-injected oocytes. *P < 0.05, **P < 0.01, and ***P < 0.001.

To explore the mechanism of LRRK2 regulating actin in oocytes, we performed MS analysis and found that several important actin-related proteins such as the Arp2/3 complex, Fascin, cofilin, and profilin were associated with LRRK2 (Figure 4A). Further verification by co-immunoprecipitation (co-IP) showed that LRRK2 was correlated with Fascin and ROCK (Figure 4B). Immunofluorescence staining showed that ROCK expression was significantly decreased at the spindle periphery in mouse oocytes after LRRK2 depletion (Figure 4C and D; 1, *n* = 36 vs. 0.71 ± 0.04, *n* = 31, *P* < 0.05). Western blotting results showed that the protein level of ROCK was significantly decreased after LRRK2 depletion (1 vs. 0.64 ± 0.04, *P* < 0.05; Figure 4E). Meanwhile, we examined cofilin, which is a downstream effector of ROCK, and indicated that phosphorylated cofilin (p-cofilin) expression was significantly decreased in the LRRK2 siRNA-injected oocytes (1 vs. 0.73 ± 0.01, *P* < 0.01; Figure 4F). Fascin could gather F-actin to form a bundle, and cofilin could affect actin polymerization of Fascin-bundled filaments. We then examined Fascin expression in the LRRK2 siRNA-injected oocytes and showed that the protein level of Fascin was also significantly decreased after LRRK2 depletion (1 vs. 0.16 ± 0.01, *P* < 0.001; Figure 4G). These results indicated that LRRK2 was essential for the expression of ROCK, p-cofilin, and Fascin during oocyte maturation.

### LRRK2 regulates mitochondrial functions in mouse oocytes

**Figure 5:**
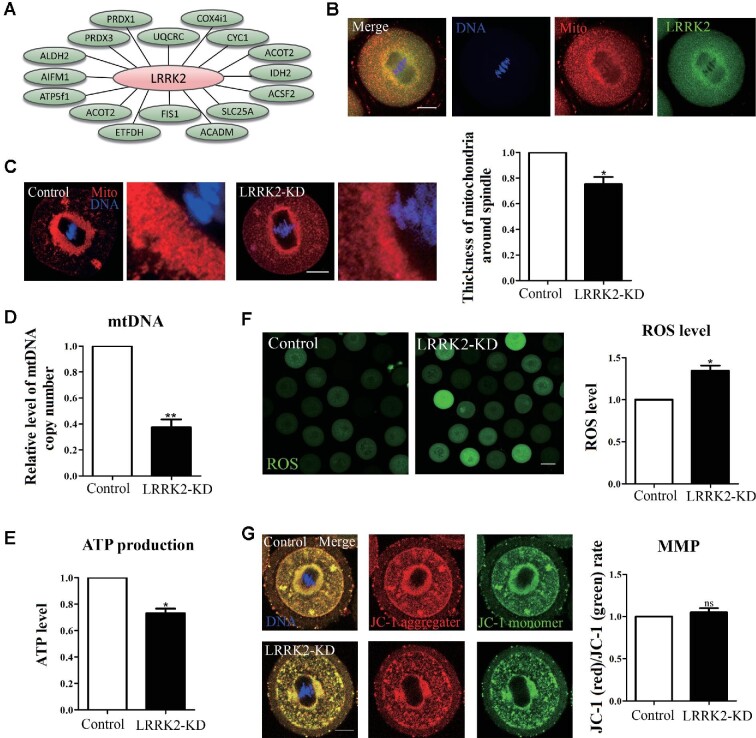
LRRK2 depletion affects the distribution and function of mitochondria in mouse oocytes. (**A**) Screening mitochondria-related proteins associated with LRRK2 by MS analysis. (**B**) LRRK2 showed a similar localization pattern to mitochondria in mouse oocytes. Red, mitochondria; green, LRRK2; blue, DNA; scale bar, 20 μm. (**C**) The accumulation of mitochondria around the spindle in the LRRK2 siRNA-injected oocytes was decreased compared with that in the control oocytes. Red, mitochondria; blue, DNA; scale bar, 20 μm. (**D**) The relative content of mtDNA copy number in the LRRK2 siRNA-injected oocytes was significantly decreased compared with that in the control oocytes. (**E**) The relative content of ATP in the LRRK2 siRNA-injected oocytes was significantly decreased compared with that in the control oocytes. (**F**) The relative fluorescence intensity of ROS in the LRRK2 siRNA-injected oocytes was significantly increased compared with that in the control oocytes. Scale bar, 50 μm. (**G**) JC-1 staining showed no significant difference in MMP between the LRRK2 siRNA-injected and control oocytes. Red, JC-1 aggregates; green, JC-1 monomer; blue, DNA; scale bar, 20 μm. *P < 0.05, **P < 0.01, and ns, no significant difference.

Actin filaments play important roles in regulating mitochondrial distribution and function. Since our MS analysis also showed close relationships between LRRK2 and several mitochondria-related proteins (Figure 5A), we then performed co-staining of mitochondria with LRRK2 and demonstrated that mitochondria were distributed around the spindle, in a similar localization pattern as LRRK2 (Figure 5B). The mitochondria accumulated around the spindle in the LRRK2 siRNA-injected oocytes were decreased compared with those in the control oocytes (1, *n* = 32 vs. 0.75 ± 0.06, *n* = 30, *P* < 0.05; Figure 5C), indicating that LRRK2 regulated the distribution of mitochondria during oocyte maturation. Subsequently, we evaluated the relative mtDNA copy number in oocytes, which could reflect the number of mitochondria. Our results demonstrated that the relative mtDNA copy number was significantly decreased after LRRK2 depletion (1, *n* = 150 vs. 0.37 ± 0.06, *n*=150, *P* < 0.01; Figure 5D), which might explain the decreased mitochondria around the spindle. Next, we examined the ATP production and showed that the ATP relative content in the LRRK2 siRNA-injected oocytes was significantly lower than that in the control oocytes (1, *n* = 180 vs. 0.73 ± 0.04, *n* = 180, *P* < 0.05; Figure 5E). Mitochondria are the center of oxidative metabolism and the principal site of reactive oxygen species (ROS) production, and the ROS level would increase if mitochondrial function is disrupted. Our results showed that the ROS levels increased in the LRRK2 siRNA-injected oocytes (1, *n* = 44 vs. 1.34 ± 0.06, *n* = 51, *P* < 0.05; Figure 5F). However, there was no significant difference in the mitochondrial membrane potential (MMP) between the control and LRRK2 siRNA-injected oocytes, which was reflected by the red/green rate of the fluorescent dye JC-1 that shifts from green to red with increasing membrane potential (1, *n* = 35 vs. 1.05 ± 0.05, *n* = 32, no significant difference; Figure 5G). These results indicated that LRRK2 regulated mitochondrial distribution, mtDNA copy number, and ATP production, but not MMP, during mouse oocyte meiosis.

## Discussion

**Figure 6:**
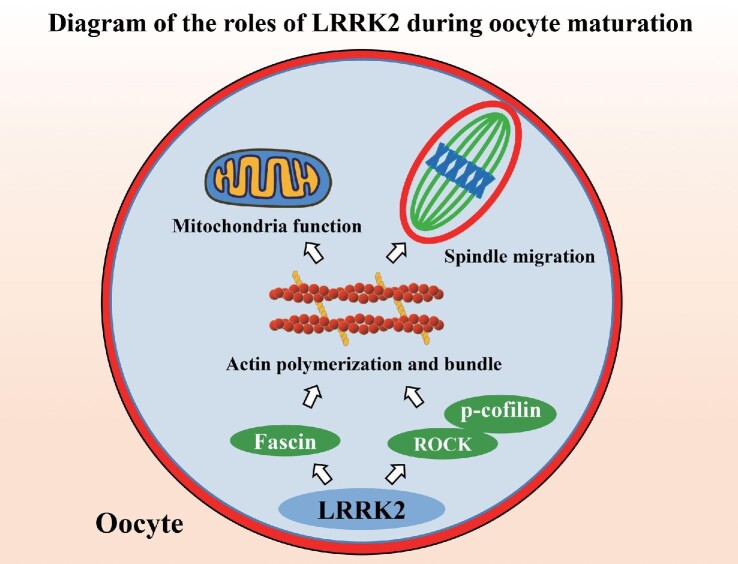
Diagram for the LRRK2 functions during mouse oocyte maturation. The cellular actin cytoskeleton is formed by the assembly of globular actin (G-actin) into double helical filaments (F-actin). LRRK2 modulates ROCK kinase and cofilin for actin assembly and Fascin for actin bundles and further affects mitochondrial function and spindle migration in mouse oocytes.

In this study, we revealed critical roles of LRRK2 in the regulation of ROCK‒cofilin- and Fascin-based actin filaments for mitochondrial function and spindle migration during oocyte meiosis (Figure 6).

LRRK2 is enriched around the spindle periphery, which is similar to cytoplasmic actin in oocytes. This localization pattern is different from somatic cells, since LRRK2 is known to be predominantly distributed throughout the cytoplasm (West et al., 2005). Previous studies also indicated that the possible subcellular locations of LRRK2 include mitochondria, endosomes, and lysosomes (West et al., 2005; Biskup et al., 2006). Moreover, LRRK2 has a similar distribution to actin-related protein RAC1 in human embryonic kidney 293 FT cells (Chan et al., 2011). The localization pattern of LRRK2 in mouse oocytes is similar to that of several reported GTPase and kinase proteins, which is consistent with the fact that LRRK2 shares the characteristics of both GTPase and kinase proteins. In mammalian oocytes, the localization of several GTPase proteins, such as RAB35 (Zhang et al., 2019a), RAB7 (Pan et al., 2020), CDC42 (Zhang et al., 2017), and RAC1 (Song et al., 2016), is reported to be related to actin filaments, while the kinase proteins, such as ROCK and LIMK, also show a similar localization pattern to actin filaments (Duan et al., 2014, 2018b). Therefore, LRRK2 may play a role in actin during oocyte meiotic maturation.

To investigate the functions of LRRK2, we disrupted LRRK2 by RNAi in mouse oocytes. LRRK2 knockdown caused two phenotypes: polar body extrusion defects and the occurrence of a large polar body. In mouse oocytes, actin is important for cytokinesis-based polar body extrusion and spindle migration-based polar body size control during oocyte meiotic maturation (Levi et al., 2011; Almonacid et al., 2014). Based on the localization pattern of LRRK2 with actin in oocytes, we first examined the distribution of actin after LRRK2 siRNA injection. Our results showed that LRRK2 depletion caused a decrease in cytoplasmic actin. LRRK2 is reported to regulate actin filaments in many models, e.g. LRRK2 regulates the F-actin/G-actin ratio of Schlemm's canal endothelium in a rat glaucoma model (Yan et al., 2020). LRRK2 binds to the actin-regulatory protein that is known to control the actin cytoskeleton responsible for myeloid cell mobility (Moehle et al., 2015). Moreover, LRRK2 is closely related to the organization of actin, which is crucial for neuronal development and neuron function (Meixner et al., 2011). In mammalian oocytes, several GTPases and kinases (LRRK2 has the GTPase and kinase domains), such as RhoA, Cdc42, RAB11a, and ROCK, are also reported to be involved in actin assembly for asymmetric division (Duan and Sun, 2019). Meanwhile, our results showed that LRRK2 depletion caused spindle migration defects at the MI stage of oocytes. Cytoplasmic actin is the main force for the mediation of spindle migration in mouse oocytes (Almonacid et al., 2014). Moreover, the dynamics of cytoplasmic actin networks regulated by vesicles can also affect spindle migration (Holubcova et al., 2013). There is a biphasic model for MI spindle migration, suggesting that formin2-mediated cytoplasmic actin assembly exerts an initial pushing force on the spindle in the first phase (Yi et al., 2013b). Based on previous reports and our results, we conclude that LRRK2 may regulate cytoplasmic actin-mediated spindle migration in mouse oocytes.

To explore the potential mechanism of LRRK2 affecting actin assembly, we performed MS analysis and showed several actin-related proteins relevant to LRRK2. Co-IP and the following western blotting results indicated that LRRK2 interacted with ROCK‒cofilin and Fascin in mouse oocytes. ROCK is an effector of RhoA GTPase, and it regulates actin assembly through its phosphorylation of cofilin and further affects oocyte meiotic maturation (Duan et al., 2014). Fascin could organize actin filaments into a cross-linked network of bundles (Li et al., 2010), while cofilin could affect actin polymerization of Fascin-bundled filaments in a concentration-dependent manner (Breitsprecher et al., 2011). Previous studies have shown that LRRK2 could regulate actin filaments through many actin-related proteins in different models. LRRK2 can regulate the phosphorylation of cofilin via PKA‒cofilin for actin dynamics and further modulates synaptogenesis and dopamine receptor activation (Parisiadou et al., 2014). LRRK2 is also critical for WASP family verprolin-homologous protein 2 phosphorylation and stability, and the concomitant increases of actin polymerization in macrophages (Kim et al., 2018). Moreover, LRRK2 mutation leads to a decreased level of RAC1 activation, which causes disassembly of actin filaments and neurite retraction (Chan et al., 2011). Our results indicate that LRRK2 may regulate ROCK‒cofilin and Fascin for actin assembly during oocyte meiotic maturation.

The distribution and function of mitochondria is a determinant of developmental competence of oocytes (Bavister and Squirrell, 2000). Our results showed that LRRK2 had a similar distribution to that of mitochondria and LRRK2 depletion affected the distribution and functions of mitochondria in mouse oocytes, evidenced by the increased ROS level, aberrant mtDNA copy number, and reduced ATP production. This may be due to the decrease of cytoplasmic actin: actin shows a close relationship to mitochondria, and actin could regulate the process of mitochondrial fission dynamics. INF2-mediated actin polymerization stimulates mitochondrial inner membrane constriction and division (Korobova et al., 2013; Chakrabarti et al., 2018). Myosin II could promote mitochondrial constriction by inducing stochastic deformations of the actin network, which takes pressure onto the mitochondrial surface (Yang and Svitkina, 2019). Actin-related mitochondrial fission is associated with mitochondrial copy number regulation, while Fascin, a prometastatic actin-bundling protein, regulates the homeostasis of mitochondrial DNA by remodeling actin (Lin et al., 2019). During oocyte meiotic maturation, actin filaments are also shown to play a vital role in maintaining the distribution and function of mitochondria (Kim et al., 2020). RAB7 GTPase regulates actin dynamics for DRP1-mediated mitochondrial function in mouse oocyte meiosis (Pan et al., 2020). Cytoplasmic actin is important for the accumulation of mitochondria at the spindle periphery, and mitochondria could provide the counterbalance for the spindle migration (Duan et al., 2020). LRRK2 is involved in many functions of mitochondria, such as mitochondrial oxidative stress, mitochondrial fission and fusion, and mitophagy (Singh et al., 2019). In yeast, LRRK2 protects against oxidative stress depending on mitochondrial function (Pereira et al., 2014). LRRK2-induced mitochondria fragmentation could be completely restored by dominant-negative DLP1 in SH-SY5Y cells (Wang et al., 2012). Moreover, LRRK2 removes damaged mitochondria by forming a complex with MIRO in induced pluripotent stem cell-derived neurons (Hsieh et al., 2016). Therefore, our results indicate that LRRK2 could affect actin-dependent mitochondrial distribution and function in mouse oocytes.

In summary, our study reveals that LRRK2 is a critical regulator for actin-based mitochondrial function and spindle migration in mouse oocyte meiosis.

## Materials and methods

### Antibodies and chemicals

Rabbit monoclonal anti-Fascin (ab126772) antibody, rabbit monoclonal anti-ROCK (ab134181) antibody, and rabbit monoclonal anti-LRRK2 antibody (ab133474) were purchased from Abcam. Rabbit monoclonal anti-GAPDH antibody (5174S), rabbit monoclonal anti-p-cofilin antibody (3313S), and mouse monoclonal anti-actin antibody (3700S) were purchased from Cell Signaling Technology. Mouse anti-α-tubulin fluorescein isothiocyanate (FITC) monoclonal antibody (F2168) was purchased from Sigma. Rabbit polyclonal Cy5-conjugated ROCK antibody (bs-1166R-Cy5) was purchased from Bioss. Tetrametrylrhodarnine isothiocyante (TRITC)-conjugated goat anti-rabbit immunoglobulin G (IgG; ZF-0516) and FITC-conjugated goat anti-rabbit IgG (ZF-0314) antibodies were purchased from Zhongshan Golden Bridge Biotechnology. Horseradish peroxidase-conjugated goat anti-rabbit IgG (H+L) antibody (CW0103) and mouse IgG (H+L) antibody (CW0102) were purchased from CWBIO. Phalloidin-TRITC was purchased from Invitrogen (R415).

### Oocyte collection and culture

The Animal Research Institute Committee of Nanjing Agricultural University approved the experimental protocols. Our research was specifically commended by this committee. GV-stage oocytes were obtained from 4-week-old ICR mouse ovaries and cultured in M2 medium (Aibei) surrounded by liquid paraffin oil (Dewin) at 37°C in a 5% CO_2_ atmosphere. The oocytes were cultured for 0, 4, 9, and 12 h after release from IBMX, corresponding to GV, GVBD, MI, and MII stages, respectively.

### Microinjection of LRRK2 siRNA

For knockdown of endogenous LRRK2, 10 pl of 50 μM LRRK2 siRNA was microinjected into the GV oocyte cytoplasm by an Eppendorf FemtoJet (Eppendorf AG) with an inverted microscope (Olympus IX53). After injection, the oocytes were cultured for 20‒24 h in M2 medium containing 200 μM IBMX (Sigma), and then washed six times in fresh M2 medium and cultured in M2 for the following experiments. The LRRK2-siRNA (F, 5′-GCAUCUCGCUGCUGAGAAATT-3′; R, 5′-UUUCUCAGCAGCGAGAUGCTT-3′) was diluted with diethypyrocarbonate (DEPC) water (GenePharma). DEPC water was microinjected as a control.

### RT-PCR analysis

RT-PCR was utilized to analyze the LRRK2 mRNA knockdown efficiency. Fifty oocytes were collected to extract total RNA by a Dynabeads mRNA DIRECT kit purchased from Invitrogen. The first strand complementary DNA (cDNA) was generated with the PrimeScript RT Master Mix (TaKaRa), and the conditions were 37°C for 15 min, 85°C for 5 sec, and 4°C. The LRRK2 cDNA fragment was amplified using the following primers: F, 5′-ATCTCACCCTTCATGCTTTCTG-3′; R, 5′-TCTCAGGTCGATTGTCTAAGACT-3′ (Genewiz). The RT-PCR reaction conditions were as follows: 95°C, 30 sec; 95°C, 10 sec and 60°C, 30 sec, for 40 cycles. The fold change in LRRK2 relative expression levels was calculated by comparing with GAPDH using the comparative 2^–ΔΔCt^ method. The GAPDH primers were as follows: F, 5′-AGGTCGGTGTGAACGGATTTG-3′; R, 5′-TGTAGACCATGTAGTTGAGGTCA-3′ (Genewiz).

### Evaluation of mtDNA copies

Total DNA of 50 oocytes was extracted by using a DNA extraction kit (Sangon) and subjected to RT-PCR analysis to evaluate the relative content of mtDNA copy number. The β-actin primers were as follows: F, 5′-TGTGACGTTGACATCCGTAA-3′; R, 5′-GCTAGGAGCCAGAGCAGTAA-3′. The MT primers were as follows: F, 5′-CCAATACGCCCTTTAACAAC-3′; R, 5′-GCTAGTGTGAGTGATAGGGTAG-3′ (Genewiz).

### Immunofluorescence and confocal microscopy

Thirty oocytes prepared for immunofluorescence staining were fixed in 4% paraformaldehyde in phosphate-buffered saline (PBS) for 30 min and permeabilized in 0.5% Triton X-100 in PBS for 20 min at room temperature. Sequentially, oocytes were blocked in 1% bovine serum albumin in PBS for 1 h at room temperature to avoid nonspecific binding. For LRRK2 staining, oocytes were incubated with a rabbit polyclonal anti-LRRK2 antibody (1:50) at 4°C overnight and subsequently washed with wash buffer (0.1% Tween 20 and 0.01% Triton X-100 in PBS) three times after incubation with the primary antibody. Next, oocytes were labelled with the secondary antibody coupled to TRITC-conjugated goat anti-rabbit IgG (1:100) at room temperature for 1 h. For α-tubulin and ROCK staining, anti-α-tubulin-FITC antibody (1:100) and Cy5-conjugated ROCK polyclonal antibody were used for incubation at 4°C for at least 7 h. Phalloidin-TRITC was used for F-actin staining and Hoechst 33342 (blue) for chromosome staining in oocytes at room temperature for 1 h. Oocytes were attached on glass slides and captured with a confocal laser-scanning microscope (Zeiss LSM 900 META). To calculate the rate of spindle migration to the cortex, the control and LRRK2 siRNA-injected oocytes were fixed after 9 h release from IBMX, and the *L* (distance from spindle pole to oocyte cortex)/*D* (oocyte diameter) ratio was analyzed.

### Quantification of the fluorescence intensity

The samples of control and LRRK2 siRNA-injected oocytes were mounted on the same glass slide, and the same setting was used for the scanning by confocal microscope. Image J software (NIH) was used to analyze the fluorescence intensity. For mitochondria and MMP staining, oocytes were cultured to the MI stage, then transferred to M16 medium with 2 μM Mito-tracker Red CMXRos (Invitrogen) and 2 μM MitoProbe JC-1 Assay Kit (Invitrogen) for 30 min, and immediately imaged under the confocal microscope.

### Assessment of ROS level

Thirty oocytes at the MI stage of the control and LRRK2 siRNA-injected groups were incubated in M2 medium containing 10 pM DCFH-DA (Beyotime Institute of Biotechnology) for 30 min at 37°C in a 5% CO_2_ incubator. Then, oocytes were transferred to a live cell-imaging dish and immediately observed by a confocal laser-scanning microscope (Zeiss LSM 900 META).

### Western blotting analysis

A total of 200 mouse oocytes were put into 10 μl NuPAGE LDS Sample Buffer (ThermoFisher Scientific) and heated at 100°C for 10 min to prepare denatured proteins. Proteins were detached by NuPAGE 10% Bis-Tris gel (160 V, 1 h) and then electrophoretically transferred to polyvinylidene fluoride membranes (20 V, 1.5 h). After transfer, the membrane was blocked in Tris-buffered saline with Tween-20 (TBST) containing 5% nonfat milk for 1 h and incubated with rabbit monoclonal anti-LRRK2 (1:5000), rabbit polyclonal anti-GAPDH (1:1000), rabbit monoclonal anti-ROCK (1:1000), rabbit monoclonal anti-p-cofilin (1:1000), rabbit monoclonal anti-Fascin (1:1000), and mouse monoclonal anti-actin (1:1000) antibodies at 4°C overnight. After washing five times in TBST (7 min each), membranes were incubated at room temperature for 1 h with horseradish peroxidase-conjugated goat anti-rabbit/mouse IgG (H+L) antibodies (1:5000). Finally, bands were imaged by an ECL Plus Western Blotting Detection System (Tanon). The band intensity values were analyzed by Image J software.

### ATP content analysis

For ATP content assessment, 30 oocytes were measured using a Bioluminescent Somatic Cell Assay Kit (Sigma). Cell ATP releasing reagent was diluted with sigma water before use. It could increase membrane permeability, and the ATP in oocytes was released immediately. Then, the treated sample was mixed with an ATP assay mix, which contains luciferase, and measured for the bioluminescence value by a multimode microplate reader (TecanSpark) to obtain the relative content of ATP.

### Co-IP and MS analysis

For co-IP analysis, 20 ovaries were put into lysis reagent containing a protease inhibitor cocktail (Cwbio). Rabbit monoclonal anti-LRRK2 antibody was mixed with the cell lysate products at 4°C overnight. Dynabeads Protein G (ThermoFisher Scientific) was added to the mixture at 4°C for 5 h, and then the tube was put to a magnet. After three washes of the bead‒antibody‒antigen complex, the immunoprecipitate was released from the beads by mixing in 2× sodium dodecyl sulfate loading buffer for 20 min at 30°C. Elution products were supplemented with NuPAGE LDS Sample Buffer (ThermoFisher Scientific) and heated at 100°C for 10 min. The input sample was ovarian lysate without antibody. For MS analysis, 500 μl of the bead‒antibody‒antigen complex was sent to GeneCreate for analysis.

### Statistical analysis

For each analysis, at least three biological replicates were performed. The data were expressed as mean ± SEM and analyzed by Student's *t*-test with GraphPad Prism 5 software. The level of significance was considered as *P* < 0.05.

## Notes

**Author contributions:** Z.-N.P. and S.-C.S. designed the experiments. Z.-N.P. performed the majority of the experiments. J.-C.L., J.-Q.J., and Y.W. contributed the reagents and materials. Z.-N.P. and S.-C.S. wrote the paper. All the authors approved the submission of this paper.
